# Burden of Lesser-Known Unintentional Non-Fatal Injuries in Rural Bangladesh: Findings from a Large-Scale Population-Based Study

**DOI:** 10.3390/ijerph16183366

**Published:** 2019-09-12

**Authors:** Lamisa Ashraf, Priyanka Agrawal, Aminur Rahman, Shumona Sharmin Salam, Qingfeng Li

**Affiliations:** 1 MPH 2019 Graduate, Johns Hopkins Bloomberg School of Public Health; 615 N Wolfe Street, Baltimore, MD 21205, USA; 2 International Injury Research Unit, Department of International Health, Johns Hopkins Bloomberg School of Public Health, 615 N Wolfe Street, Baltimore, MD 21205, USA; 3 Center for Injury Prevention and Research, Bangladesh, House B 162, Rd No. 23, Dhaka 1206, Bangladesh; 4 International Center for Diarrheal Disease Research, Bangladesh, 68, Shaheed Tajuddin Ahmed Sarani Mohakhali, Dhaka 1212, Bangladesh

**Keywords:** cut injuries, unintentional poisoning, machine injuries, injury by blunt objects, Bangladesh

## Abstract

Around 90% of all fatal and non-fatal unintentional injuries occur in low- and middle-income countries (LMICs). The magnitude of unintentional injuries is unclear due to limited research and data. This paper describes the burden of lesser-known injuries (LKIs—cut injuries, unintentional poisoning, machine injuries, electrocution, injury by blunt objects, and suffocation) in rural Bangladesh, using data from the Saving of Lives from Drowning (SoLiD) project in Bangladesh. Descriptive statistics were used to report counts and rates of injuries by socio-demographic factors, injury characteristics, and circumstantial details. The annual morbidity rate of LKIs was 6878 injuries per 100,000 persons, involving 3.4% (40,520) of the population. Cut injury (44,131.2/100,000 per year) and injury by blunt objects (19768.6/100,000 per year) attributed in large numbers to the overall burden of LKIs. Males (66.1%) suffered more injuries than females. More than half (52.9%) occurred among people aged 25 to 64 years. Those involved in agriculture suffered the most injuries, mainly cut injuries (9234.1/100,000 per year) and machine-related injuries (582.9/100,000 per year). Most injuries occurred in the home setting. Increased awareness about packaging, storage, and the proper handling of appliances can help lower the frequency of LKIs. Safe architecture and awareness about home injuries is required to reduce injuries occurring in the home environment.

## 1. Introduction

There has been a rise in the rate of unintentional injuries globally, claiming more than 25.62 lives per 100,000 people in 2017 alone [1]. With every unintentional injury death, there are many times more non-fatal injuries that result in the loss of school and work days and financial costs in treatment and rehabilitation, which are difficult to bear, particularly by people in low- and middle-income countries (LMICs). The global burden of mortality and morbidity due to unintentional injuries has been a challenge to deal with over the years as many of these injuries go unreported due to lack of awareness, scarcity of vital registration and population-based data, and improper record keeping in hospitals in LMICs [2,3]. Additionally, many injured persons do not attend hospitals for treatment in LMICs [3]. The lack of awareness is particularly true for lesser-known unintentional injuries like cut injuries, injuries by blunt objects, electrocution, unintentional poisoning, machine injuries, and suffocation, which pose a problem across the world. However, more than 90% of these fatal and non-fatal injuries occur in LMICs [2,4].

Lesser-known injuries impose a heavy burden. Industrialization and lack of awareness towards easily accessible household supplies contributed to about 86,400 deaths globally in 2015 in the form of unintentional poisoning [5]. Excessive consumption of alcohol and drug use contributed to a large part of the Disability-Adjusted Life Years (DALYs) due to unintentional poisoning in males [5]. Similarly, urbanization has created more jobs transitioning from manual to machine, making machine injuries a top occupational injury with almost a six-fold increase in deaths among people 20 to 70 years of age [6]. Over half of the fatal injuries occurring in farmers, foresters, fishers, and injuries in over one-third of the crafts workers are due to machinery. In rural areas of LMICs, where electricity is increasingly being used in homes and for agricultural purposes, non-fatal electrocution injuries are quite common [7].

Bangladesh is no exception to the shifting paradigm of urbanization and rapid development. In 2017 alone, lesser-known unintentional injuries, namely poisoning, suffocation, cut injuries, and electrocution, led to a vast number of deaths and morbidities in Bangladesh [1]. Cut injuries are very common as most of the household and agriculture-based work is manual in nature. The suffering and disability resulting from the inadequate management of these injuries leads to increased hospitalizations and work loss days, leading to economic constraints [8,9].

The aim of this paper is to describe the socio-demographic characteristics and injury characteristics of non-fatal cut injuries, unintentional poisoning, machine injuries, electrocution, injury by blunt objects, and suffocation, using data from a census covering approximately 1.18 million people in seven rural sub-districts of Bangladesh. 

## 2. Methods

### 2.1. Data Collection and Procedure

The data used for this analysis was collected as part of the Saving of Lives from Drowning (SoLiD) project in Bangladesh across 51 unions from seven sub-districts of the country in 2013. The SoLiD project was rolled out with the main objective of evaluating the large-scale effectiveness and cost effectiveness of two drowning prevention interventions in reducing drowning deaths in children under five years of age [10]. The SoLiD baseline survey collected general demographic and socio-economic information, household and environmental characteristics pertaining to drowning, as well as information on all external causes of injury, including but not limited to drowning, cuts, falls, suicide, injury by blunt objects, and others. The study used the opportunity of a large-scale sample size to generate baseline statistics for injuries on a population level that had not been collected in the region before.

A baseline census with a total population of approximately 1.18 million respondents from all households in 51 unions was conducted in 2013. The data included information on the socio-demographic and injury-related characteristics of all included individuals. Fatal and non-fatal injury events were recorded with a recall period of one year and six months, respectively. For the purposes of this study, an injury event was defined when a person sought treatment or had to miss one work or school day due to the injury [2]. 

The data collection included two rounds of interviews—the first round collected socio-demographic information as well as the occurrence of an injury, the second round focused only on those households that reported an injury event within the follow-up period. The second round of interviews involved the use of injury modules (see injury modules in Supplementary Materials), which collected circumstantial details of the specific type of injury event. All tools in the study were pre-defined and pre-tested. Interviews were conducted in the native language by trained data collectors, who collected information from household heads or any adult above 18 years of age by face-to-face interviews. Interviews were supervised by field supervisors, who observed 10% of the interviews, repeated interviews for 2% of the households every day and checked 10% of the forms, selected randomly, to ensure the quality of data collected [2]. A codebook for all the questions was developed prior to data collection and all variables of interest were recorded in codes on the data collection forms. In this paper, we focused on the unintentional non-fatal injury events related to cut injuries, unintentional poisoning, machine injuries, electrocution, injuries by blunt objects, and suffocation. Other injury causes, such as suicide, road traffic injuries, violence, falls, burns, and drowning, that shared the major burden of injuries in rural Bangladesh have been discussed separately [2,11,12,13,14,15]. 

### 2.2. Statistical Analysis

Descriptive statistical analyses were conducted to obtain the counts of non-fatal events and morbidity rates per 100,000 persons per year (with 95% confidence intervals), disaggregated by socio-demographic characteristics such as sex, age, education, occupation, marital status, and sub-district. Since non-fatal events were recorded over a period of six months, the rates of non-fatal events were annualized by doubling the rates for six months with the assumption that the injury rates were constant throughout the year. Sex was coded as a binary variable and age was categorized into eight groups. Education, occupation, and marital status were categorized as nominal categorical variables with seven, nine, and six categories, respectively. The circumstances of each injury type were described in detail. Stata 15.1 I/C package was used for all statistical analyses [16]. Approval for this study was obtained from Institutional Review Boards of the Johns Hopkins Bloomberg School of Public Health, Center for Injury Prevention and Research, Bangladesh (CIPRB), and International Center for Diarrheal Disease Research, Bangladesh (ICDDR, B). 

## 3. Results

Our study population was comprised of 51.5% females (Table 1). Around 43.4% of the respondents in the study population were in the 25–64 years age group. The age and sex distribution in the dataset were representative of the population of Bangladesh [2]. Around 34.9% of the study participants had a primary level of formal education and 35% were either retired or unemployed. In the total sample population, 3.4% of people suffered the six non-fatal injuries included in this paper with an overall morbidity rate of 6877.9 injuries per 100,000 per year (95% CI: 6872.5–6944.1). 

### 3.1. Cut Injuries 

A total of 26,311 (22.1%) people suffered from cut injuries in the study population with a morbidity rate of 4466.1/100,000/year. The morbidity rate of cut injuries was significantly higher for males (5815.2/100,000/year (95% CI: 5728.8–5902.8)) than for females (3187.9/100,000/year (95% CI: 3125.4–3251.7)) (Table 1). People with no education (5274.0/100,000/year (95% CI: 5159.9–5390.7)) had the highest rates of cut injuries. Those involved in agriculture had more cut injuries than other occupations with a morbidity rate of 9234.1/100,000/year. Most occurrences of cut injuries took place in the home setting (a single living space compartmentalized into separate functional areas) and its surrounding areas (56%) (Table 2).

The object that caused the most cut injuries was a “boti/da” (37.9%). A boti is a sharp cutting instrument with a long blade that cuts on a platform held down by the foot. A boti/da is used for cutting fruits, vegetables, meat, etc. in rural households and is regularly used during cooking. Other objects such as a sickle, broken glass, scissors, and a knife were also associated with cut injuries. In 65.3% of cases, the injured person was working with the sharp instrument when the injury occurred (Table 3). 

### 3.2. Unintentional Poisoning 

Females (*n* = 45; 51.7%) had a slightly higher number of occurrences of unintentional poisoning than males (*n* = 42; 48.3%) (Table 1). People with advanced degrees having more than 15 years of education (42.3/100,000/year), children aged 1–4 years (65.4/100,000/year), and transport workers (46.1/100,00/year) had the highest morbidity rates for unintentional poisoning. Pesticides (28.7%) were the most common product associated with unintentional poisoning, followed by medicines (24.5%) and kerosene (13.8%). Other poisons used were insecticides (*n* = 2), rodenticides (*n* = 8), sleeping pills (*n* = 3), and soap and detergent (*n* = 10). More than 80% of poisonings occurred in the home environment (Table 2). A bottle was used to store chemicals 50% of the time, whereas products were kept in original packets 21% of the time.

### 3.3. Machine Injuries

The morbidity rate of machine injuries was 396.0/100,000/year (95% CI: 373.6–419.7) and 50.9/100,000/year (95% CI: 43.5–59.6) for males and females, respectively. Skilled professionals had more non-fatal machine injuries (*n* = 374; 29.0%) compared to people in other occupations. The highest number of machine injuries occurred in people who had primary level education (*n* = 466; 36.2%). Married people had the highest occurrences of machine injuries (*n* = 864; 67.0%) (Table 1). Machines were mainly used for agricultural, industrial, or construction purposes. Among agricultural machines, shallow machines such as pumps caused the greatest number of injuries (34.9%). 

### 3.4. Electrocution

The morbidity rate of electrocution was slightly higher in males (175.1/100,000/year) than in females (116.7/100,000/year). High numbers of electrocutions were seen in retired and unemployed people (*n* = 259; 30.3%), those with primary level education (*n* = 311, 36.4%), and among those who were married (*n* = 463; 54.2%) (Table 1). The source of electricity for most people who had an electrocution event was inside the home (73.7%), specifically in the bedroom. Outside the home environment, electrocution injuries occurred more commonly in markets (26.7%). 

### 3.5. Injuries by Blunt Objects

As seen with other injuries, injuries by blunt objects were also more common in males (2919.6/100,000/year; 95% CI: 2858.2–2982.4) than in females (1129.9/100,000/year; 95% CI: 1092.7–1168.3). Unskilled and domestic workers (5566.5/100,000/year), people with no formal education (2197.3/100,000/year), and married people (2325.3/100,000/year) had more injuries by blunt objects than others (Table 1). Fixed blunt objects caused 61.6% of injuries and occurred most commonly in households (*n* = 4398; 37.3%) followed by construction sites (*n* = 2132; 18.1%). 

### 3.6. Suffocation

Suffocation injuries were more common in females (35.0/100,000/year) than in males (30.0/100,000/year). Individuals with primary education (*n* = 70; 36.5%), retired/unemployed (*n* = 97; 50.5%), and married people (*n* = 122; 63.5%) had more suffocation injuries than others. Retired/unemployed persons suffered more from suffocation injuries (Table 1). Most suffocation injuries occurred in homes and surroundings (Table 2). Suffocation was most commonly caused by choking on food items (52.2%). Around 9% of suffocating injuries occurred when the individual’s face got covered by clothes, plastic materials, an adult body, or by earth while playing. 

## 4. Discussion

In this study, unintentional injuries were more common in males than in females, except for unintentional poisoning and suffocation, which were higher in females. The non-fatal injury events were highest in the 25–64 years age group for both males and females, for most injuries, except unintentional poisoning, which was more common in children 1–4 years of age. For electrocution, injury by blunt objects, and suffocation, there was a decreasing trend in morbidity rates with more years of formal education. Except for unintentional poisoning, non-fatal injuries were highest for married persons, compared to other categories, for all five injuries. Retired/unemployed people had the highest numbers of non-fatal electrocution and suffocation injuries. Most of the injuries (except machine injuries) occurred in the home environment and surrounding areas.

In this study, men suffered more unintentional injuries than women. A study that used the Global Burden of Disease data, along with reports from World Health Organization, United Nations Children’s Fund, and the World Bank, also found similar differences of the burden of injuries shared by men and women [17]. In rural Bangladesh, men are the primary breadwinners involved in occupational work and are exposed to appliances and machines that put them at a higher risk of such injuries [18].

Unintentional poisoning was highest in children under five years of age, as has been seen in other LMIC contexts [19]. Previous studies from Bangladesh have shown ingestion of pesticides, kerosene, and paraffin to be quite common [17]. These products are readily available in households for disinfection and cooking purposes, however, they are inadequately stored [5,17,20]. Very young children have an inherent exploratory and inquisitive nature that puts them at a higher risk of exposure to such unsafe products. Additionally, children can get attracted to the packaging, as has been seen in Sri Lanka where kerosene is sold in colorful packets [19]. Moreover, these chemicals are usually stored in juice or other drink bottles in rural homes and are easily confused with beverages [19]. Limited literacy among elderly women and lack of attention from family members might explain the high proportions of unintentional poisoning events in this group of the population. The general public needs to be made aware of using appropriate containers to store household products and providing supervision to very young children as well as the elderly. Manufacturing companies could childproof their products, and print hazard indicators in the local language as well as pictorial diagrams on the containers to account for the lack of reading skills [19].

Agricultural machines were shown to contribute more to machine-related injuries in our study as well as in studies from India [21,22]. Studies from LMICs have revealed that farmers use minimal protective wear while working, which exposes them to more injuries [23]. There is a need to train farmers in the correct and safe use of machines, make protective clothing available for farmers, and set up first aid response systems to address injuries sooner to prevent long-term disabilities [24]. The heavy reliance on manual labor with the slow introduction of automatic machinery in rural Bangladesh increases the risk of blunt object injuries. For example, a study showed that most agricultural injuries occurred as a result of tools slipping from workers’ hands and handling devices manually [22]. Non-compliance to safety precautions and lack of adequate training to handle tools is also a reason cited in research for increased blunt object injuries [22].

In this study, electrocution, like other injuries, occurred more commonly among men compared to women, and mostly at home. Males are more likely to get involved in fixing broken or interrupted electric cables and other appliances that put them at a higher risk of electrocution [25]. Children should also be supervised and should not play with or near electric cables. The fact that most electrocutions occurred in the home suggests the improper or careless use and application of electric wiring, fittings, and cords [26].

Reviewing the gap in the literature about consistent and evidence-based interventions for managing such injuries, it would be safe to at least suggest awareness and education programs for communities to improve their literacy about these less common injuries and methods to reduce and manage injuries. To raise awareness and promote safety for the prevention of injuries, a study in China, via government funding, introduced the concept of “Safe Communities”—a platform for high-risk and vulnerable groups as well as others across age and gender in the community to engage and promote injury safety literacy [27]. Local governments can be advocated to reallocate funds in rural parts of the country to create these communities and bring local villagers together to discuss injuries and prevention strategies. Even with the SoLiD project, village injury prevention communities were set up and led by local government members to discuss different injuries, their prevention strategies, as well as strategies to involve the overall community in raising awareness. There is scope to scale up such initiatives across other parts of the country as a promising measure to reduce the burden of unintentional injuries in Bangladesh and other LMICs.

Existing literature gives information about the prevention of road traffic injuries, drowning, falls, burns, etc. and about several policies established to prevent such injuries, such as swimming pool fencing, seatbelt use, window bars to prevent falls, and the use of smoke-detectors, respectively, for example [28]. However, there is a gap in the literature regarding the means to prevent the injuries discussed in this paper. Studies discuss how certain policies from high-income countries might be suitable for LMICs while some need to be modified entirely [28]. In LMICs, in addition to policies and research, resources and financial support are necessary, as well as community engagement, for buying into the policies and projects targeted to reduce injuries [28]. Furthermore, the approach to dealing with the high burden of injuries in LMICs should be multidisciplinary [28].

There are some limitations to the study. As a six-month recall period was used for collecting data on non-fatal injuries, recall bias may be present in our data. As the prevalence of deaths from the six injuries was very low, we focused only on non-fatal injury events, thereby underreporting the true burden of unintentional injuries in rural Bangladesh. Additionally, data were collected only from rural Bangladesh, thus results may not be generalizable to urban Bangladesh as risk factors and lifestyles vary. Additionally, the specific injuries need to be studied in more detail to understand their implications and develop targeted interventions to reduce their respective burden. 

## 5. Conclusions

The purpose of this study was to provide urgently needed insights into the burden of lesser-known injuries, which attribute to a significant number of morbidities. It is evident that these injuries are a common occurrence in the households of rural Bangladesh. Education and awareness programs to make safer homes can tackle the burden of these injuries. Further studies are needed to investigate the short-term and long-term implications of such injuries on lives and livelihoods in rural Bangladesh. Based on the study results and identified gaps in data, we propose developing a multidisciplinary intervention package to raise awareness and improve safety measures to confront the burden of lesser-known injuries, and integrating injury prevention programs in existing national surveillance platforms. 

## Figures and Tables

**Table 1:** Socio-demographic characteristics of the study population for different injuries in rural Bangladesh.

Socio-Demographic Characteristics	*N* (%) *N* * = 1,178,256	Non-Fatal Injuries (n, %, Morbidity Rate/100,000/year) *n* ** = 40,520 (3.4%, 6877.9)											
		Cut Injuries		Unintentional Poisoning		Machine Injuries		Electrocution		Injury by Blunt Objects		Suffocation	
		*n*	Rate	*n*	Rate	*n*	Rate	*n*	Rate	*n*	Rate	*n*	Rate
Total		26,311	4466.1	87	14.8	1289	218.8	855	145.1	11,786	2000.6	192	32.6
Sex													
Males	567,674 (48.5)	16,667	5815.2	42	14.7	1135	396.0	502	175.1	8368	2919.6	86	30.0
Females	601,919 (51.5)	9644	3187.9	45	14.9	154	50.9	353	116.7	3418	1129.9	106	35.0
Age (years)													
<1	22,141 (1.9)	70	648.3	0	0.0	0	0.0	0	0.0	24	222.3	1	9.3
1–4	90,523 (7.7)	1682	3667.0	30	65.4	33	71.9	41	89.4	550	1199.1	12	26.2
5–9	139,728 (12.0)	3111	4412.0	8	11.3	64	91.0	77	109.2	1017	1442.3	13	18.4
10–14	142,121 (12.2)	2979	4160.4	3	4.2	67	93.6	98	136.9	1310	1829.5	8	11.2
15–17	62,098 (5.3)	1173	3748.8	0	0.0	69	220.5	49	156.6	665	2125.3	8	25.6
18–24	133,534 (11.4)	2395	3560.4	4	5.9	193	286.9	107	159.1	1268	1885.0	11	16.4
25–64	508,059 (43.4)	13,660	5312.4	37	14.4	822	319.7	443	172.3	6354	2471.1	107	41.6
65+	71,389 (6.1)	1241	3580.7	5	14.4	41	118.3	40	115.4	598	1725.4	32	92.3
Education													
No education	295,314 (25.3)	7815	5274.0	22	14.8	385	259.8	200	135.0	3256	2197.3	64	43.2
Primary	407,923 (34.9)	10,325	5010.4	20	9.7	466	226.1	311	150.9	4478	2173.0	70	34.0
Secondary	289,658 (24.8)	5626	3851.9	12	8.2	363	248.5	261	178.7	2979	2039.6	39	26.7
A levels	45,618 (3.9)	628	2740.0	1	4.4	28	122.2	32	139.6	384	1675.4	3	13.1
College	13,526 (1.2)	130	1913.2	1	14.7	13	191.3	7	103.0	99	1457.0	3	44.2
Advanced/Professional degree	4729 (0.4)	30	1269.0	1	42.3	1	42.3	3	126.9	15	634.5	0	0.0
Not applicable (U5 children)	112,664 (9.6)	1752	3091.8	30	52.9	33	58.2	41	72.4	574	1013.0	13	22.9
Occupation													
Agriculture	104,956 (9.0)	4879	9234.1	5	9.5	308	582.9	93	176.0	1595	3018.7	23	43.5
Business	61,661 (5.3)	1326	4275.8	3	9.7	103	332.1	62	199.9	797	2570.0	9	29.0
Skilled labor (Professional)	89,151 (7.6)	2486	5509.2	8	17.7	374	828.8	129	285.9	1792	3971.2	10	22.2
Unskilled/domestic (Unskilled)	24,520 (2.1)	882	7094.9	0	0.0	86	691.8	23	185.0	692	5566.5	8	64.4
Rickshaw/bus (Transport worker)	17,037(1.5)	447	5146.8	4	46.1	69	794.5	18	207.3	296	3408.2	1	11.5
Students	312,537 (26.7)	6127	3890.9	9	5.7	140	88.9	213	135.3	2784	1767.9	28	17.8
Retired/unemployed/housewife	408,583 (35.0)	7563	3679.2	26	12.6	148	72.0	259	126.0	3010	1464.3	97	47.2
Not applicable (children)	144,454 (12.4)	2483	3414.6	32	44.0	59	81.1	53	72.9	791	1087.8	16	22.0
Not applicable (others)	5948 (0.5)	101	3464.8	0	0.0	2	68.6	4	137.2	22	754.7	0	0.0
Marital status													
Married	571,206 (48.8)	14,845	5151.4	40	13.9	864	299.8	463	160.7	6701	2325.3	122	42.3
Never married	227,319 (19.4)	4307	3762.8	6	5.2	282	246.4	193	168.6	2484	2170.2	21	18.3
Divorced	3220 (0.3)	67	4317.1	0	0.0	4	247.0	3	185.2	21	1296.7	0	0.0
Widowed	53,096 (4.5)	841	3205.1	2	7.6	17	64.8	36	137.2	430	1638.8	18	68.6
Separated	2717 (0.2)	51	3719.9	0	0.0	1	72.9	2	145.9	29	2115.2	0	0.0

* *N* represents the total number of people in the study population. ** *n* represents the total number of injured persons in the study population. 95% CIs of the morbidity rates were included in Appendix 1 (Supplementary Materials).

**Table 2:** Characteristics of non-fatal injuries by place of injury.

Place of Injury	Injuries *n* = 40,520 (%)					
	Cut Injuries	Unintentional Poisoning	Machine Injuries	Electrocution	Injury by Blunt Objects	Suffocation
Home and surroundings ^1^	14,634 (56.0)	70 (80.0)	291 (23.0)	625 (73.0)	4777 (41.0)	168 (88.0)
Place of education ^2^	589 (2.0)	0	3 (0)	14 (2.0)	1476 (13.0)	2 (1.0)
Roads/Highway	1451 (6.0)	3 (3.0)	88 (7.0)	33 (4.0)	1418 (12.0)	9 (5.0)
Railway, water reservoir, ferry, launch station	1006 (4.0)	2 (2.0)	33 (3.0)	6 (1.0)	385 (3.0)	1 (1.0)
Agricultural field	6382 (24.0)	8 (9.0)	386 (30.0)	41 (5.0)	1230 (10.0)	2 (1.0)
Industry/Factory/Workshop	393 (1.0)	1 (1.0)	307 (24.0)	33 (4.0)	479 (4.0)	0
Others ^3^	1856 (7.0)	3 (3.0)	181 (14.0)	103 (12.0)	2021 (17.0)	10 (5.0)

^1^ Home and surroundings comprised of bedroom, living room, kitchen, bathroom, yard, veranda, one-room dwelling. ^2^ Place of education comprised of school or other playground, hostel or academic institute, and classroom. ^3^ Others comprised of market, office, construction (with its categories), others, and unknown.

**Table 3:** Circumstances of cut injuries.

Circumstances of Cut Injuries *n* = 26,311	*n*	%
Type of sharp object that cut the person		
Boti/Da	9985	37.9
Sickle	2996	11.4
Broken glass	2818	10.7
Scissors	2314	8.8
Knife	581	2.2
How the person was injured with the object		
Working	17,188	65.3
Fell on the object	4794	18.2
Playing	3578	13.6
Place where the sharp object was usually stored		
Bedroom	8914	33.9
Kitchen and Dining area	8522	32.4
Single-room dwelling	977	3.7
Storage room	937	3.6
Veranda	873	3.3
Living area	374	1.4
Bathroom	251	1.0

## Supplementary Materials

Supplementary materials are available online at https://www.mdpi.com/1660-4601/16/18/3366/s1. Appendix 1: 95% CIs for the morbidity rates of different injuries for each socio-demographic characteristic. Injury modules used during the second round of interviews were also included as supplementary materials.
