# Supplementary material for: Burden of Lesser-Known Unintentional Non-Fatal Injuries in Rural Bangladesh: Findings from a Large-Scale Population-Based Study
Source: Int J Environ Res Public Health. 2019 Sep 12;16(18):3366. doi: 10.3390/ijerph16183366 (PMC6766074; doi:10.3390/ijerph16183366)
Supplement: Supplementary file 1 [file ijerph-16-03366-s001.zip › Supplemental_files_-_Lesser-known_injuries.docx]

Supplemental files

**APPENDIX**

Appendix 1: 95% CIs for morbidity rates of different injuries for each socio-demographic characteristic

| **Socio-demographic characteristics** | **Non-fatal injuries (95% Confidence intervals for morbidity rates)** | | | | | |
| --- | --- | --- | --- | --- | --- | --- |
|  | **Cut injuries** | **Unintentional poisoning** | **Machine injuries** | **Electrocution** | **Injury by blunt objects** | **Suffocation** |
| **Total** | **4413.0 – 4519.8** | **12.0-18.2** | **207.2- 231.1** | **135.7-155.2** | **1965.0-2036.8** | **28.3-37.5** |
| **Sex** |  |  |  |  |  |  |
| Males | 5728.8- 5902.8 | 10.8- 19.8 | 373.6- 419.7 | 160.5- 191.2 | 2858.2- 2982.4 | 24.3-37.1 |
| Females | 3125.4- 3251.7 | 11.1- 19.9 | 43.5- 59.6 | 105.1- 129.5 | 1092.7- 1168.3 | 29.0- 42.4 |
| **Age (years)** |  |  |  |  |  |  |
| <1 | 513.1- 819.1 | . | . | . | 149.0- 331.5 | 1.3-65.7 |
| 1-4 | 3497.3- 3844.7 | 45.7- 93.5 | 51.1- 101.2 | 65.8- 121.4 | 1103.2- 1303.2 | 14.9-46.1 |
| 5-9 | 4261.3- 4568.0 | 5.7- 22.7 | 71.0- 116.0 | 87.3- 136.5 | 1356.6- 1533.4 | 10.7- 31.8 |
| 10-14 | 4015.1- 4310.9 | 1.4- 13.0 | 73.6- 118.9 | 112.3- 166.8 | 1733.5- 1930.8 | 5.6-22.3 |
| 15-17 | 3542.1- 3967.3 | . | 174.2- 279.2 | 118.4- 207.2 | 1970.5- 2292.1 | 12.8-51.1 |
| 18-24 | 3421.8- 3704.5 | 2.2- 15.8 | 249.2- 330.3 | 131.6- 192.2 | 1784.5- 1991.1 | 9.1-29.5 |
| 25-64 | 5225.3- 5401.1 | 10.4- 19.9 | 330.3- 342.3 | 157.0- 189.1 | 2411.4- 2532.2 | 34.4-50.3 |
| 65+ | 3388.5- 3783. 6 | 6.0- 34.7 | 87.1- 160.6 | 84.7- 157.3 | 1593.0- 1868.7 | 65.3-130.5 |
| **Education** |  |  |  |  |  |  |
| No education | 5159.9- 5390.7 | 9.8- 22.5 | 235.1- 287.1 | 117.5- 155.0 | 2123.5- 2273.7 | 33.8-55.2 |
| Primary | 4915.9- 5106.8 | 6.3 – 15.0 | 206.5- 247.6 | 135.0- 168. 7 | 2110.7- 2237.3 | 26.9-42.9 |
| Secondary | 3753.4- 3952.8 | 4.7- 14.5 | 224.2- 275.4 | 158.3 -201.7 | 1968.0- 2113.8 | 19.5-36.5 |
| A levels | 2535.1- 2961. 2 | 0.6- 31.0 | 84.4- 176.9 | 98.7- 197.4 | 1516.5- 1850.8 | 4.2-40.6 |
| College | 1612.1- 2269.8 | 2.1- 104.5 | 111.1- 329.4 | 49.1- 216.0 | 1197.1 – 1772.7 | 14.2- 136.9 |
| Advanced/Professional Degree | 887.9- 1812.3 | 6.0- 300.1 | 6.0- 300.1 | 40.9- 393.2 | 382.7- 1051.3 | . |
| Not applicable (U5 children) | 2951.4- 3238.8 | 37.0- 75.7 | 41.4- 81.9 | 53.3- 98.3 | 933.6- 1099.1 | 13.3-39.5 |
| **Occupation** |  |  |  |  |  |  |
| Agriculture | 8984.3- 9490.4 | 3.9- 22.7 | 521.4- 651.7 | 143.7-215.7 | 2875.2- 3169.3 | 28.9- 65.5 |
| Business | 4053.9- 4509.5 | 3.1- 30.0 | 273.8- 402.8 | 155.9- 256.4 | 2398.6- 2753.4 | 15.1- 55.8 |
| Skilled labor (Professional) | 5299.6- 5726.8 | 8.9- 35.4 | 749.1- 917.0 | 240.6- 339.7 | 3793.2- 4157.4 | 11.9-41.2 |
| Unskilled/domestic (Unskilled) | 6649.1- 7569.4 | . | 560.2- 854.2 | 123.0- 278.3 | 5171.8- 5990.4 | 32.2-128.7 |
| Rickshaw/bus (Transport worker) | 4696.2- 5639.4 | 17.3- 122.7 | 627.7- 1005.3 | 130.6- 328.8 | 3043.9- 3815.3 | 1.6-81.7 |
| Students | 3795.6- 3988.5 | 3.0-11.0 | 75.3- 104.9 | 118.3- 154.7 | 1703.7- 1834.5 | 12.3-25.8 |
| Retired/unemployed/housewife | 3597.9- 3762.3 | 8.6- 18.6 | 61.3- 84.6 | 111.6- 142.3 | 1413.1- 1517.3 | 38.7-57.6 |
| Not applicable(children) | 3284.0- 3550.4 | 31.1- 62.2 | 62.9- 104.7 | 55.7- 95.4 | 1014.7- 1166.1 | 13.5-35.9 |
| Not applicable(others) | 2854.8- 4202.5 | . | 17.2- 274.2 | 51.5- 365.4 | 497.1- 1145.0 | . |
| **Marital status** |  |  |  |  |  |  |
| Married | 5070.2- 5233.8 | 10.2- 18.9 | 280.5- 320.5 | 146.7- 176.0 | 2270.6- 2381.3 | 35.5-50.6 |
| Never married | 3653.1- 3875.8 | 2.4- 11.7 | 219.2- 276.8 | 146.4-194.2 | 2086.9- 2256.7 | 12.0-28.1 |
| Divorced | 3262.2- 5240.4 | . | 92.7- 657.4 | 59.7- 573.8 | 846.1- 1985.0 | . |
| Widowed | 2997.2 – 3427.2 | 1.9- 30.5 | 40.3- 104.2 | 99.0- 190.2 | 1491.5- 1800.4 | 43.2-108.9 |
| Separated | 2832.2- 4879.0 | . | 10.3- 517.3 | 36.5- 582.7 | 1471.6- 3036.1 | . |
