# Supplementary material for: Burden of Lesser-Known Unintentional Non-Fatal Injuries in Rural Bangladesh: Findings from a Large-Scale Population-Based Study
Source: Int J Environ Res Public Health. 2019 Sep 12;16(18):3366. doi: 10.3390/ijerph16183366 (PMC6766074; doi:10.3390/ijerph16183366)
Supplement: Supplementary file 1 [file ijerph-16-03366-s001.zip › injury modules/M-10 electrocution.docx]

| **Saving of Lives from Drowning (SoLiD)**  **ICDDR,B and CIPRB Baseline Survey/Injury Surveillance** | | | | | | |
| --- | --- | --- | --- | --- | --- | --- |
| Gg 10-we`y¨Z¯ú„‡÷i Z_¨  **M 10–Electrocution** | | | | | | |
|  | |  | |  | | |
|  | | **bvg Name** | | **†KvW Code** | | |
| **Dc‡Rjv** Upazila | |  | |  | | |
| **BDwbqb** Union | |  | |  | | |
| eø­K Block | |  | |  | | |
| **MÖvg** Village | |  | |  | | |
| **Lvbvi b¤^i** Household no. | |  | | / | | |
| **Lvbv cÖav‡bi bvg** Name of Household Head | |  | |  | | |
| **ZvwiL** Date | |  | | **Y**  **M**  **M**  **Y**  D  **D**D | | |
|  | |  | |  | | |
| No. | Questions | | Coding Categories | | | Skip |
| 1. | e¨w³i bvgName of person | | ________________________________________ | | |  |
| 2. | **e**¨w³i Lvbv m`m¨ b¤^iPerson Number | |  | | |  |
| 3. | we`y¨‡Zi Drm wK wQj ?  What was the source of electricity? | | eRªcvZ Lighting…………………………………………..…  evox‡Z e¨eüZ we`y¨r Electric using inside home………………  evoxi evwn‡i e¨eüZ we`y¨r Electric using outside of home……  KviLvbvq e¨eüZ we`y¨r Electric using in a factory …………...  evwo ev KviLvbv e¨ZxZ wewìsG e¨eüZ we`y¨r…………………….  Electric using in a building other than house or factory | | 1  2  3  4  5 | Q 05 |
| 4. | hw` evox‡Z nq, †Kv_vq N‡UwQj ?  If inside home, where did the electrocution occur? | | ivbœv Ni Kitchen………………………………………...…...  emvi Ni Living area …………………………………….....  †kvqvi Ni Bedroom…………………………………………  GK K¶ wewkó Ni Single room dwelling ……………..…….  †MvmjLvbv/cvqLvbv Bathroom…………………………….…..  eviv›`v Veranda……………………………………………...  Ab¨vb¨ (D‡jøL Kiæb) Others (Specify) ______________....... | | 1  2  3  4  5  6  7 |  |
| 5. | hw` evoxi evwn‡i nq, †Kv_vq we`¨yr¯ú„ó n‡qwQj?  If outside home, where did the electrocution occur? | | iv¯Ívq Street………………………………………………….  gv‡V Field …………………………………………………...  Awd‡m Office…………………………………………………  wkí cÖwZôv‡b Factory/Industry……………………………  nvU/evRvi Market……………………………………………  Ab¨vb¨ (D‡jøL Kiæb) Others (Specify)______________ | | 1  2  3  4  5  6 | END |
