# Supplementary material for: Burden of Lesser-Known Unintentional Non-Fatal Injuries in Rural Bangladesh: Findings from a Large-Scale Population-Based Study
Source: Int J Environ Res Public Health. 2019 Sep 12;16(18):3366. doi: 10.3390/ijerph16183366 (PMC6766074; doi:10.3390/ijerph16183366)
Supplement: Supplementary file 1 [file ijerph-16-03366-s001.zip › injury modules/M-13 blunt objects.docx]

| **Saving of Lives from Drowning (SoLiD)**  **ICDDR,B and CIPRB Baseline Survey/Injury Surveillance** | | | | | | |
| --- | --- | --- | --- | --- | --- | --- |
| Gg 13- †fuvZv e¯‘ Øviv AvNvZ  **M 13 – Blunt Object** | | | | | | |
|  | |  | |  | | |
|  | | bvg **Name** | | †KvW **Code** | | |
| Dc‡Rjv Upazila | |  | |  | | |
| BDwbqb Union | |  | |  | | |
| eø­K Block | |  | |  | | |
| MÖvg Village | |  | |  | | |
| Lvbvi b¤^i Household No | |  | | / | | |
| Lvbv cÖav‡bi bvg Name of Household Head | |  | |  | | |
| ZvwiL Date | |  | | **Y**  **M**  **M**  **Y**  D  **D**D | | |
|  | |  | |  | | |
| No. | Questions | | Coding Categories | | | Skip |
| 1 | e¨w³i bvg Name of person | | ________________________________________ | | |  |
| 2 | e¨w³i Lvbv m`m¨ b¤^i Person Number | |  | | |  |
| 3 | †fuvZv e¯‘wU wK wQj ?  What was the blunt object? | | PjšÍ e¯‘ (bvg wjLyb) Moving objects (specify)………………..  w¯’i e¯‘ (bvg wjLyb) Fixed objects (specify)………………......  Ab¨vb¨ (D‡jø¨L Kiæb) Others (Specify)_________________ | | 1  2  9 |  |
| 4 | †fuvZv e¯‘wU wK Kv‡R e¨envi Kiv nw”Qj ?  What was the blunt object used for? | | Pvlvev`/Lvgv‡ii Kv‡R Farm……………………………….…  M„n¯’vjxi Kv‡R Household…………………………………..  Kj-KviLvbvi Kv‡R Factory…………………………………  †`vKv‡bi Kv‡R Shop…………………………………………  wbg©vY Kv‡R Construction……………………………….……  Awdm Av`vj‡Z Office………………………………………  †Ljva~jvq Game………..........................................................  Ab¨vb¨ (D‡jøL Kiæb) Others (Specify)_____________ | | 1  2  3  4  5  6  7  9 | END |

Code of the blunt object: names
