# Supplementary material for: Burden of Lesser-Known Unintentional Non-Fatal Injuries in Rural Bangladesh: Findings from a Large-Scale Population-Based Study
Source: Int J Environ Res Public Health. 2019 Sep 12;16(18):3366. doi: 10.3390/ijerph16183366 (PMC6766074; doi:10.3390/ijerph16183366)
Supplement: Supplementary file 1 [file ijerph-16-03366-s001.zip › injury modules/M-14 suffocation.docx]

| **Saving of Lives from Drowning (SoLiD)**  **ICDDR,B and CIPRB Baseline Survey/Injury Surveillance** | | | | | | |
| --- | --- | --- | --- | --- | --- | --- |
| Gg 14- k¦vm‡iva  **M 14 – Suffocation** | | | | | | |
|  | |  | |  | | |
|  | | bvg **Name** | | †KvW **Code** | | |
| Dc‡Rjv Upazila | |  | |  | | |
| BDwbqb Union | |  | |  | | |
| eø­K Block | |  | |  | | |
| MÖvg Village | |  | |  | | |
| Lvbvi b¤^i Household No | |  | | / | | |
| Lvbv cÖav‡bi bvg Name of Household Head | |  | |  | | |
| ZvwiL Date | |  | | **Y**  **M**  **M**  **Y**  **D**  **D**D | | |
|  | |  | |  | | |
| No. | Questions | | Coding Categories | | | Skip |
| 01 | **e¨w³i bvg** Name of person | | ________________________________________ | | |  |
| 02 | **e¨w³i Lvbv m`m¨ b¤^i** Person Number | |  | | |  |
| 03 | **k¦vm‡iv‡ai KviY?**  What was the suffocating agent? | | Kve©b g‡bv·vBW Carbon monoxide……………………..…...  Ab¨ M¨vm (D‡jøL Kiæb) Other gas (specify)………………….  Zij(D‡jøL Kiæb) Liquid (specify)…………….....................  gv‡Qi KuvUv Fishbone………………………………………...  Ab¨ Lv`¨e¯‘(D‡jøL Kiæb) Other food items (specify)………..  avZe gy`ªv (cqmv/UvKv) Coin……………………………….....  †QvU e¯‘ (D‡jøL Kiæb) Other Small object (specify)…………  Kvc‡o †X‡K hvIqvi Kvi‡Y Covered by clothes………………  cøvwó‡Ki e¨vM/`ªe¨ Øviv †X‡K hvIqv……………………………..  Covered by plastic bags/ materials  eo e¨w³i kix‡ii †Kvb As‡ki Pv‡c Covered by adult body…..  gvwU Pvcv covq Covered by earth…………………………...  Ab¨vb¨ (D‡jøL Kiæb) Others (Specify) _____________ | | 1  2  3  4  5  6  7  8  9  10  11  12 |  |
| 04 | **NUbvi mswÿß (m‡e©v”P wZbwU ev‡K¨) weeib**  Describe in three sentences what happened? | | …………………………………………………………………………………………………………………………………………………………………………………………………………………………………………………………………………………………………………… | |  | End |
