# Supplementary material for: Burden of Lesser-Known Unintentional Non-Fatal Injuries in Rural Bangladesh: Findings from a Large-Scale Population-Based Study
Source: Int J Environ Res Public Health. 2019 Sep 12;16(18):3366. doi: 10.3390/ijerph16183366 (PMC6766074; doi:10.3390/ijerph16183366)
Supplement: Supplementary file 1 [file ijerph-16-03366-s001.zip › injury modules/M-5 cut injury.docx]

| **Saving of Lives from Drowning (SoLiD)**  **ICDDR,B and CIPRB Baseline Survey/Injury Surveillance** | | | | | | |
| --- | --- | --- | --- | --- | --- | --- |
| Gg 5-aviv‡jv e¯‘i AvNvZ  **M 5–Cut injury** | | | | | | |
|  | |  | |  | | |
|  | | **bvg Name** | | **†KvW Code** | | |
| **Dc‡Rjv** Upazila | |  | |  | | |
| **BDwbqb** Union | |  | |  | | |
| **ø­K** Block | |  | |  | | |
| **MÖvg** Village | |  | |  | | |
| **Lvbvi b¤^i** Household No. | |  | | / | | |
| **Lvbv cÖav‡bi bvg** Name of Household Head | |  | |  | | |
| **ZvwiL** Date | |  | | **Y**  M  **M**  **Y**  **D**  **D** | | |
|  | |  | |  | | |
| No. | Questions | | Coding Categories | | | Skip |
| 1. | e¨w³i bvg Name of person | | ________________________________________ | | |  |
| 2. | e¨w³i Lvbv m`m¨ b¤^i Person Number | |  | | |  |
| 4. | aviv‡jv e¯‘wU wK wQj ?  What was the sharp object that cut the person? | | Qywi Knife…………………………………………………….  `v/ewU Boti/Da……………………………………………….  Kv‡¯Í Sickle………………………………………………….  KuvwP Scissors…………………………………………………  KvuPfv½v Broken glass………………………………………..  Ab¨vb¨ (D‡jøL Kiæb) Others (Specify)_____________ _____ | | 1  2  3  4  5  9 |  |
| 5. | e¨w³wU H e¯‘ Øviv wK KiwQj ?  What was the person doing with that object? | | KvR Working………………………………………………..  †Ljv Playing…………………………………………………  e¯‘wUi Ic‡i c‡o wM‡qwQj Fell on the object…………………Ab¨vb¨ (D‡jøL Kiæb) Others (specify)………………………. | | 1  2  3  9 |  |
| 6. | mvaviYZt KvR †k‡l aviv‡jv e¯‘wU †Kv_vq ivLv nq ?  Where is the sharp object usually stored? | | ivbœvNi Kitchen………………………………………………  †kvqvi Ni Bedroom………………………………….……….  LvIqvi NiDining area……………………………………….  emvi Ni Living area…………………………………………  †MvmjLvbv/cvqLvbv Bathroom……………………………….  †÷vi iæg/¸`vg NiStorage room……………………………..  GK K¶ wewkó NiSingle room dwelling………………………  eviv›`v Veranda……………………………………………..  Ab¨vb¨ (D‡jøL Kiæb) Others (Specify) _____________ | | 1  2  3  4  5  6  7  8  9 | END |
