# Supplementary material for: Burden of Lesser-Known Unintentional Non-Fatal Injuries in Rural Bangladesh: Findings from a Large-Scale Population-Based Study
Source: Int J Environ Res Public Health. 2019 Sep 12;16(18):3366. doi: 10.3390/ijerph16183366 (PMC6766074; doi:10.3390/ijerph16183366)
Supplement: Supplementary file 1 [file ijerph-16-03366-s001.zip › injury modules/M-8 unintentional poisoning.docx]

| **Saving of Lives from Drowning (SoLiD)**  **ICDDR,B and CIPRB Baseline Survey/Injury Surveillance** | | | | | | |
| --- | --- | --- | --- | --- | --- | --- |
| **Gg 8-`yN©UbvRwbZ welwµqv**  **M 8 –Unintentional Poisoning** | | | | | | |
|  | |  | |  | | |
|  | | **bvg Name** | | **†KvW Code** | | |
| Dc‡Rjv Upazila | |  | |  | | |
| BDwbqb Union | |  | |  | | |
| eø­K Block | |  | |  | | |
| MÖvg Village | |  | |  | | |
| Lvbvi b¤^i Household no. | |  | | / | | |
| Lvbv cÖav‡bi bvg Name of Household Head | |  | |  | | |
| ZvwiL Date | |  | | **Y**  **M**  **M**  **Y**  D  **D**D | | |
|  | |  | |  | | |
| No. | Questions | | Coding Categories | | | Skip |
| 01. | e¨w³i bvg Name of person | | ________________________________________ | | |  |
| 02. | e¨w³i Lvbv m`m¨ b¤^i Person Number | |  | | |  |
| 04. | we‡li cªKvi Categories  we‡li aiY wK wQj ?  What was the type of poison? | | KxUbvkK Pesticides…………………………………….......  gkv, kvwQ, †Zjv‡cvKv BZ¨vw` aŸsmKvix Insecticides………….  Bu`yi gviv wel Rodenticides……………………………...... .  Ny‡gi ewo Sleeping pills…………….....................................  Ab¨vb¨ Jla Other medicine………………………………..  Mvevb/¸uov mvevb Soap/detergent……….….............................  †K‡ivwmb Kerosene…………...……………………………...  m¨vfjb, †WUj Savlon/Dettol………………………………….  Ab¨vb¨ (D‡jøL Kiæb) Others (Specify)....................................  Rvbv †bB Don’t know …………………………..…….......... | | 1  2  3  4  5  6  7  8  9  99 |  |
| 05. | wK ai‡bi cv‡Î wel ivLv n‡qwQj ?  In what type of container was the poison kept? | | †evZjBottle……………………………………………........  †KŠUv Container ……………………………………………..  c¨v‡KU Packet………………………………………………..  wóªc Strip………………………………………………….....  Ab¨vb¨ (D‡jøL Kiæb) Others (Specify)……………………… | | 1  2  3  4  5 |  |
| 06. | cv‡Îi cÖK…wZ Nature of container  cvÎwU wK cÖK…Z bv Ab¨ wQj ?  Was the container original? | | cÖK…Z Original……………………………………………......  Ab¨ Not original…………………………………………….. | | 1  2 | Q 08 |
| 07. | hw` Ab¨ cvÎ nq Z‡e wK †mUv †j‡ej Kiv wQj ?  If not original, was it labeled? | | †j‡ej Kiv Labelled…………………………………………  †j‡ej wenxb Not labelled…………………………………… | | 1  2 |  |
| 08. | hw` †evZj nq Z‡e cv‡Îi gyL eÜ wQj wK ?  If bottle, was the container capped? | | nu¨v Yes………………………………………………………  bv No………………………………………………………..  Rvbv †bB Don’t know……………………………………… | | 1  2  9 |  |
| 09. | cvÎwU †Kv_vq ivLv n‡qwQj ? (¯’vb)  Where the container was kept (place)? | | †g‡S‡Z Floor…………………………………………........  LvU/†PŠwKi bx‡P Under bed…………………………………..  †ZvlK/ cvwUi wb‡P Under mattress…………………………...  ev‡·i g‡a¨ Inside box………………………………….........  †mj&d Gi Dc‡i (1 wgUv‡ii D‡×©)……………...........................  Shelf (>1 m from the floor)  †mj&d Gi Dc‡i (1 wgUv‡ii bx‡P )………………………………  Shelf (< 1m from the floor)  wmwjs G Syj¯Í Ae¯’vq Hanging from ceiling…………………..  gvPvq SzjšÍ Ae¯’vq Bamboo shelf (*macha*)………………..…  Ab¨vb¨ (D‡jøL Kiæb) Others (Specify)………………………. | | 1  2  3  4  5  6  7  8  9 |  |
| 10. | cvÎwU wK Ae¯’vq wQj ?  What was the status of the container? | | †Lvjv Ae¯’vq Open place…………………………………......  Zvjve× Ae¯’vq Locked…………………………………….... | | 1  2 | END |
