# Supplementary material for: Burden of Lesser-Known Unintentional Non-Fatal Injuries in Rural Bangladesh: Findings from a Large-Scale Population-Based Study
Source: Int J Environ Res Public Health. 2019 Sep 12;16(18):3366. doi: 10.3390/ijerph16183366 (PMC6766074; doi:10.3390/ijerph16183366)
Supplement: Supplementary file 1 [file ijerph-16-03366-s001.zip › injury modules/M-9 machine injuries.docx]

| **Saving of Lives from Drowning (SoLiD)**  **ICDDR,B and CIPRB Baseline Survey/Injury Surveillance** | | | | | | |
| --- | --- | --- | --- | --- | --- | --- |
| Gg 9- †gwkb/hš¿cvwZi Øviv RLg  **M 9–Machine/tool injury** | | | | | | |
|  | |  | |  | | |
|  | | **bvg Name** | | **†KvW Code** | | |
| **Dc‡Rjv** Upazila | |  | |  | | |
| **BDwbqb** Union | |  | |  | | |
| **ø­K** Block | |  | |  | | |
| **MÖvg** Village | |  | |  | | |
| **Lvbvi b¤^i** Household no. | |  | | / | | |
| **Lvbv cÖav‡bi bvg** Name of Household Head | |  | |  | | |
| **ZvwiL** Date | |  | | **Y**  **M**  **M**  **Y**  D  **D**D | | |
|  | |  | |  | | |
| No. | Questions | | Coding Categories | | | Skip |
| 1. | e¨w³i bvg Name of person | | ________________________________________ | | |  |
| 2. | e¨w³i Lvbv m`m¨ b¤^i Person Number | |  | | |  |
| 3. | GUv wK ai‡bi †gwkb wQj ?  What type of machine caused the injury? | | K…wl Kv‡R e¨eüZ †gwkb Agricultural…………………….........  wkí KviLvbvq e¨eüZ †gwkb Industrial……………………….  wbg©vY Kv‡R e¨eüZ †gwkb Construction………………………  Ab¨vb¨ (D‡jøL Kiæb) Others (Specify)……………………..... | | 1  2  3  4 | Q5  Q6  Q7 |
| 4. | K…wl †gwkb n‡j, wK ai‡bi †gwkb wQj ?  What is the agricultural machine that caused the injury? | | k¨v‡jv ‡gwkb I Ab¨vb¨ cv¤ú………………………………......  Shallow machine or other pumps  UªvKUi Tractor…………………………………………….…  avb gvovB Gi Kj Rice grain separating machine…………...  Ab¨vb¨ hš¿PvwjZ †gwkb Other powered machine…………….. | | 1  2  3  4 |  |
| 5. | wkí KviLvbvi †gwkb n‡j, GUv wK ai‡bi †gwkb wQj?  What is the factory / industry machine that caused the injury? | | ivBm wgj Rice milling/polishing machine………………….  †cÖm/QvcLvbv Pressing machine…………………………….  wQ`ª Kivi hš¿/wWªj †gwkb Drilling machine………………...  KivZ Kj Sawing machine…………………………………  †mjvB †gwkb Sewing machine……………………………..  I‡qwìs †gwkb Welding machine……………………………  Grinding machine…………………………………..............  Ab¨vb¨ (D‡jøL Kiæb) Others (Specify)…………………….… | | 1  2  3  4  5  6  7  8 |  |
| 6. | GUv wK ai‡bi wbg©vY Kv‡R e¨eüZ †gwkb wQj?  What is the construction machine that caused the injury? | | wg·Pvi †gwkb Mixture machine…………………………...….  fvB‡eªUi Vibrator……………………………………….……  †gvRvBK KvUvi Mosaic cutter………………………..….....  Ab¨vb¨ (D‡jøL Kiæb) Others (Specify)………………....…… | | 1  2  3  4 | END |
