# Supplementary material for: Burden of Lesser-Known Unintentional Non-Fatal Injuries in Rural Bangladesh: Findings from a Large-Scale Population-Based Study
Source: Int J Environ Res Public Health. 2019 Sep 12;16(18):3366. doi: 10.3390/ijerph16183366 (PMC6766074; doi:10.3390/ijerph16183366)
Supplement: Supplementary file 1 [file ijerph-16-03366-s001.zip › injury modules/Morbidity-revised_7th April.docx]

| **Saving of Lives from Drowning (SoLiD)**  **ICDDR,B and CIPRB Baseline Survey/Injury Surveillance** | | | | | | |
| --- | --- | --- | --- | --- | --- | --- |
| BbRywiRwbZ Amy¯’Zv wbeÜxKiY dg©  **Injury Morbidity Form** | | | | | | |
|  | |  |  | | | |
|  | | **bvg Name** | **†KvW Code** | | | |
| Dc‡Rjv Upazila | |  |  | | | |
| BDwbqb Union | |  |  | | | |
| eøK Block | |  |  | | | |
| MÖvg Village | |  |  | | | |
| Lvbvi b¤^i Household Number | |  | / | | | |
| Lvbv cÖav‡bi bvg Name of Household Head | |  |  | | | |
| ZvwiL Date | |  | **Y**  **M**  **M**  Y  **D**  **D** | | | |
| DËi`vZvi bvg I Lvbv m`m¨ b¤^i Name & household number of the respondent | |  |  | | | |
| No. | Questions | Coding Categories | | | | Skip |
| 01. | BbRywiRwbZ Amy¯’ e¨vw³i bvg  Name of injured person | ________________________________________ | | | |  |
| 02. | BbRywiRwbZ Amy¯’ e¨vw³i Lvbv m`m¨ b¤^i Injured person number |  | | | |  |
| 03. | BbRywiRwbZ Amy¯’ e¨vw³i wj½  Sex of injured person | cyiæl Male………………………………………………..............................  gwnjv Female…………………………………………………………….. | | | 1  2 |  |
| 04. | AwfcÖvq Intent  BbRywii AwfcÖvq wK wQj ?  What was the intent of the injury? | **A**wb”QvK…Z Unintentional………… ……………………………………….  B”QvK…Z wb‡Ri ¶wZ Intentional/Self harm……………..…………………..  mwnsmZv Assault/Violence…………………………………………………....  wbY©q Kiv hvqwb Undetermined…………………………………………….... | | | 1  2  3  4 |  |
| 05. | ¯’vb Place  AvNvZ cªvwßi mgq e¨w³wU †Kv_vq wQj?  Where was the person when s/he was injured? | †kvqvi Ni Bed room…………………………………………………………  emvi NiLiving room…………………………………………………..........  ivbœv Ni Kitchen……………………………………………………………  †Mvmj Lvbv/cvqLvbv Bathroom………………….…………………………….  evoxi DVvb Yard………………………………………………………........  eviv›`v Verenda …………………………………………. ………………..  GK K¶ wewkó Ni One room dwelling……………………............................  †kªYxK¶ Class room………………………………………………………..  ¯‹z‡ji †Ljvi gvV School play ground……………………………………….  wk¶v cÖwZôv‡bi †nv‡÷j Hostel of educational institute………………………  Ab¨ †Ljvayjvi ¯’vb/ µxov½b Other playground…………….............................  iv¯Ívq /gnvmo‡KRoads/highway……………………………………….........  †ij‡ókb/†dix/jÂ NvU/evm÷¨vÛ/Ab¨ cwienb GjvKv…………………………..  Railway station/Ferry/ Launch station/Bus stand/ Other vehicles area  K…wl†¶Î/Lvgvi evox/PvZvj Agricultural field…………………………………  wkí/KjKviLvbv/IqvK©kc Industry/factory/workshop………………………..  Rjvkq Water reservoir..…………………………………............................  nvU/ evRvi/ Market/Bazaar…………………………………………….........  Awdm Office………………………………………………………………..  wbg©vY GjvKv Construction area……………………………………………….  Ab¨vb¨ (D‡jøL Kiæb) Others (Specify) _________________________  Rvbv †bB Don’t Know……………………………………………………… | | | 1  2  3  4  5  6  7  8  9  10  11  12  13  14  15  16  17  18  19  20  99 |  |
| 06. | KZ Zvwi‡L BbRywi N‡UwQj ?  What was the date of injury ? | D D M M Y Y | | | |  |
| 07. | KLb BbRwyi N‡UwQj ? **(**24 N›Uvq wjLyb**)**  What was the time of injury? | H H M M | |  | |  |
| 08. | BbRywi c×wZ Injury Mechanism  wKfv‡e BbRywi n‡qwQj ?  What was the external cause of injury?  (wbw`©óBbRywi c×wZ dg©c~iY Kiæb  Please fill in the specific injury mechanism form) | AvZ¥nZ¨v-Gg 1 Attempt to suicide/suicide-M1……………………………..  moK `yN©Ubv-Gg 2 Transport injury- M2…………………..........................  mwnsmZv-Gg 3 Violence-M 3……………………………...........................  c‡o hvIqv-Gg 4 Fall-M4………………………………………………….  aviv‡jv e¯‘ Øviv †K‡U hvIqv-Gg 5 Cut injury-M-5…………………………..  cy‡o hvIqv-Gg 6 Burn-M-6……………………………………………….  Wy‡e hvIqv-Gg 7 Drowning-M-7…………………………………………..  `yN©UbvRwbZ welcvb-Gg 8 Unintentional poisoning –M-8…….........................  †gwkb/hš¿cvwZi AvNvZ-Gg 9 Machine injury -M-9….……………………..  we`~¨r¯ú„„ó -Gg 10 Electrocution-M-10……………………………………..  cÖvbx I KxU cZ‡½i Kvgo / AvNvZ-Gg 11 Animal bite injury-M-11…….......  gP‡K hvIqv/Uvb cov -Gg 12 Sprain/strain-M-12…………..........................  †fuvZv e¯‘i AvNvZ -Gg 13 Blunt object-M-13…………………………......  `yN©Ubv RwbZ k¦vm‡iva-Gg 14 Suffocation-M-14……………………………  cošÍ e¯‘i AvNvZFalling object…………………………………………….  Ab¨vb¨ (D‡jøL Kiæb) Others (Specify)____________________________  Rvbv †bBDon’t know…………………………………………………....... | | 1  2  3  4  5  6  7  8  9  10  11  12  13  14  15  16  99 | |  |
| 09. | ¶Z A½ I BbRywii aiY  Injured parts and type of injury  e¨w³wUi kix‡ii †Kvb †Kvb A‡½ Ges wK ai‡bi BbRywi n‡qwQj?  What were the injured parts and the type of injury? |  | | | |  |

|  | ¶Z A½ Injured parts BbRywii aiYInjury types (Main three) | | | | | | | | | |
| --- | --- | --- | --- | --- | --- | --- | --- | --- | --- | --- |
|  | 1. gv_v Head |  |  |  |  |  |  |  |  |  |
|  | 2. gyL gÛj Face |  |  |  |  |  |  |  |  |  |
|  | 3. Nvo Neck |  |  |  |  |  |  |  |  |  |
|  | 4. eyK/wcV Chest |  |  |  |  |  |  |  |  |  |
|  | 5. †cU Abdomen |  |  |  |  |  |  |  |  |  |
|  | 6. evû nvZ e¨ZxZ Upper extremity (except hand) |  |  |  |  |  |  |  |  |  |
|  | 7. nvZ Hand |  |  |  |  |  |  |  |  |  |
|  | 8. wbgœv½ (cv‡qi cvZv e¨ZxZ) Lower extremity (except foot) |  |  |  |  |  |  |  |  |  |
|  | 9.cv‡qi cvZv Foot |  |  |  |  |  |  |  |  |  |
|  | 10.†Kvgi Waist |  |  |  |  |  |  |  |  |  |

**BbRywi ai‡bi †KvW (Injury types):** 01. nvo fv½v(Fracture) 02.gP‡K hvIqv(Sprain) 03. nvo m‡i hvIqv(Dislocation) 04. †K‡U hvIqv / Db¥y³ ¶Z(Cut/open wound) 05. Kvgo(Bite) 06. wQ‡j/ †_uZ‡j hvIqv (Abrasion/laceration) 07.cy‡o hvIqv (Burn.) 08.gv_vq AvNvZ(Head injury) 09. kix‡ii wfZ‡ii A‡½i ¶Z (Internal injury/internal organ injury) 10. Ab¨vb¨ (D‡jøL Kiæb) (Others specify)………………………

| 10. | hLb BbRywi N‡UwQj ZLb e¨w³wUi Ae¯’v †Kgb wQj?  What was the condition of the victim just after injury | mÁvb Conscious…………………………………………….......  msÁvnxb Unconscious……………………………………….......  Rvbv †bB (Unknown)…………………………............................ | 1  2  9 | Q12  Q12 |
| --- | --- | --- | --- | --- |
| 11. | hLb Bb&&Rywi N‡UwQj, ZLb e¨w³wUi Pjv‡div Kivi ¶gZv †Kgb wQj? (hw` Ávb _v‡K)  What was the mobility condition of the person just after injury (if the person is conscious) | GKv GKv nvuU‡Z ‡c‡iwQj Mobile alone…………………………………  A‡b¨i mn‡hvwMZvq nvuU‡Z ‡c‡iwQj Mobile with assistance……………..  nvuU‡Z cviwQj bv Immobile……………………………………………..  Rvbv †bB Don’t know……………………………………………….. | 1  2  3  9 |  |
| 12. | AvnZ e¨w³wU‡K †KD wK cÖv_wgK wPwKrmv w`‡qwQj ?  Did the person receive first aid? | nu¨v Yes……………………………………………..............................  bvNo……………………………………………….............................  Rvbv †bB Don’t know…………………............................................... | 1  2  9 | Q15  Q15 |
| 13. | DËi nu¨v n‡j, †K cÖv_wgK wPwKrmv w`‡qwQj ? (GKvwaK DËi)  If yes, who gave first aid?  (Multiple responses) | gv Mother……...................................................................................  evev Father………...............................................................................  ¯^vgx-¯¿x Husband/wife………………………………………………..  dv‡g©mx/cjøx wPwKrmK Medicine shopkeeper/village doctor…………….  Ab¨ cÖvß eq¯‹ †mev`vbKvix Other adult caregiver………………….......  fvB/†evbBrother/sister……………………………………………….  eÜz / mgeqmx wkï Friend/peer…………………………………………  cÖwZ‡ekx Neighbor…………...............................................................  gvV ch©v‡qi ¯^v¯’¨Kg©x Community health worker……………………….  KwgDwbwUi ‡¯^”Qv‡mex Kg©x Community volunteer……………………..  Wv³vi/nvmcvZvj/wK¬wbK Doctor/hospital/clinic…………………….......  wbR Himself/herself……………………………………………………  Ab¨vb¨ (D‡jøL Kiæb) Others (Specify)………………...…………….… | A  BC  D  E  F  G  H  I  J  K  L  X |  |
| 14. | †m wK cÖv_wgK wPwKrmvq cÖwk¶Y cÖvß ?  Was s/he trained in first aid? | nu¨v Yes………………………………………………………………...  bv No………………………………………………………………….  Rvbv †bB Don’t know……………………………………………….. | 1  2  9 | Q16  Q16 |
| 15. | BbRywii Rb¨ †Kvb wPwKrmv MÖnb Kiv n‡qwQj wK ?  Did the person receive treatment for injury? | nu¨v Yes………………………………………………………………..  bv No………………………………………………............................  Rvbv †bB Don’t know…....................................................................... | 1  2  9 | Q27  Q27 |
| 16. | wPwKrmv cÖ`vbKvix Treatment provider  AvnZ e¨w³‡K †K wPwKrmv K‡iwQj?  Who provided the treatment? | †iwRóvW© Wv³vi Registered doctor..…....................................................  †gwW‡Kj Gwm÷¨v›U/SACMO Medical Assistant/SACMO..…..............  ¯^v¯’¨ mnKvix / cwievi Kj¨vY cwi`wk©Kv / cwievi Kj¨vY mnKvix Health Assistant/Family Welfare Visitor/Family Welfare Assistant…….…  Gb. wR. I ¯^v¯’¨ Kg©x ©NGO Service Provider……………………………  Jl‡ai †`vKvb`vi / cjøx wPwKrmK………………………………………  Medicine shopkeeper/Village doctors  †nvwgIc¨vw_K wPwKrmK Homeopathic Practitioner…………….………..  KweivR Herbal Medicine Practitioner………………………….……...  cÖwk¶Y cÖvß `vB Trained TBA……………………................................  Svo duyKKvix / Bgvg/cy‡ivwnZ …………………………………………  Traditional healer/Religious Leader  AvZ¥xq-¯^Rb / eÜz-evÜe Relative/Friends…………...............................  Ab¨vb¨ D‡jøL Kiæb Others (Specify) _____________ ­­­­­­­­­­­­­­­­­­­­­­­­­­­­­­_______________ | A  B  C  D  E  F  G  H  I    J  X |  |
| 17. | wPwKrmv cÖ`vbKvix cÖwZôvb  Facility  AvnZ e¨w³wU †Kv_vq wPwKrmv wb‡qwQj?  Where did the person receive treatment? | we‡klvwqZ nvmcvZvj †gwW‡Kj K‡jR nvmcvZvj, c½y nvmcvZvj, wc. wR. nvmcvZvj BZ¨vw`) ……………………………………………...  Specialized hospital (Medical College Hospital, Orthopaedic  Hospital, Post graduate Hospital etc)  †Rjv nvmcvZvj District Hospital…………………………………...  Dc‡Rjv ¯^v¯’¨ Kg‡cø· Upazila Health Complex………………........  BDwbqb ¯^v¯’¨ I cwievi Kj¨vY †K›`ª ………………………………..  Union Health and Family Welfare Centre  cÖvB‡fU wK¬wbK Private Clinic……………………………………...  Gb.wR.I wK¬wbK NGO Clinic………………………………………  †mev`vbKvixi cÖvB‡fU †P¤^vi Private practitioners’ chambers………  dv‡g©mx/Jl‡ai †`vKvb`vi…………………………………………..  Pharmacy/medicine shopkeeper  wbR evox‡Z Own home………………………………………….  Ab¨vb¨ (D‡jøL Kiæb) Others (Specify) _____________ ______________ | A  B  C  D  E  F  G  H  I  X |  |
| 18. | AvnZ e¨w³ nvmcvZv‡j/¯^v¯’¨†K‡›`ª fwZ© n‡qwQj wK ?  Was the injured person admitted to a health facility? | nu¨v Yes………………..........................................................................  bv No………………………………………………...........................  Rvbv †bB Don’t know……………………………………………….. | 1  2  9 |  |
| 19. | (DËi Ônu¨vÕ n‡j) AvnZ e¨w³‡K hw` nvmcvZvj/¯^v¯’¨‡K›`ª /wK¬wb‡K fwZ© Kiv n‡q _v‡K Zvn‡j †Kvb ai‡bi nvmcvZvj ?  , what type of health facility was s/he admitted to? | we‡klvwqZ nvmcvZvj (†gwW‡Kj K‡jR nvmcvZvj, c½y nvmcvZvj, wc. wR. nvmcvZvj BZ¨vw`) …………………………………………………….  Specialized hospital (Medical College Hospital, Orthopaedic Hospital, Post graduate Hospital, etc.)  †Rjv nvmcvZvj District Hospital…………………………………...  Dc‡Rjv ¯^v¯’¨ Kg‡cø· Upazila Health Complex……………………......  cÖvB‡fU wK¬wbK Private Clinic………………………………………..  Gb.wR.I wK¬wbK NGO Clinic………………………………………..  Ab¨vb¨ (D‡jøL Kiæb)Others (Specify) __________________________ | 1  2  3  4  5  9 |  |
| 20. | nvmcvZv‡j/wPwKrmv cÖ`vbKvixi wbKU wKfv‡e wb‡q hvIqv n‡qwQj ?  Transportation to hospital/other treatment provider: How was the patient transported to the health facility? | G¨v¤^y‡jÝ Ambulance……………………………………………..  Ab¨ †Kvb †gvUi PvwjZ Mvwo‡Z (evm, Rxc, Kvi Ges wZb PvKvwewkó Mvwo)  Other motorized vehicle (bus, jeep car and three wheeler………….  †gvUinxb hvbevnb (wi·v, wi·v f¨vb, †VjvMvwo) ...................................  Non-motorized vehicle (rickshaw, rickshaw van, cart, etc  †bŠKv Boat……………………………………...........................  †dix /jÂ /÷xgvi Ferry/launch/steamer…………………………....  †mev`vbKvix AvNvZcÖvß e¨w³‡K evwo‡Z †`L‡Z G‡mwQ‡jb …………….…  Treatment provider visited the injured person at home  Ab¨vb¨ (D‡jøL Kiæb) Others (Specify)__________________________  Rvbv †bB Don’t know………………………………………….. | A  B  C  D  E  F  X  Y |  |
| 21. | AvNvZ cvIqvi ci nvmcvZv‡j/†mev`vbKvixi wbKU †cuŠQv‡Z KZ mgq †j‡MwQj ? (N›UvqwjLyb) [hw` GK N›Uvi Kg nq Z‡e 000wjLyb]  How much time did it take to reach the provider? (in hours) [if less than one hour write 000] |  |  |  |
| 22. | e¨w³wU nvmcvZv‡j KZw`b fwZ© wQj ?  How many days was the person admitted in the health facility? |  |  |  |
| 23. | AvnZ e¨w³i mvR©vix ev Acv‡ikb †j‡MwQj wK?  Was surgery/operation done? | nu¨v Yes……………………………………………………………..…...  bv No………………………………………………………………….  Rvbv †bB Don’t know………………………………………………… | 1  2  9 | Q25  Q25 |
| 24. | DËi Ôn¨vuÕ n‡j Acv‡ik‡b wK ai‡bi A¨v‡bm&‡_wmqv †`qv n‡qwQj?  If yes, what type of anesthesia was given? | †jvKvj Local………………………………………………………….  mvaviY General……………………………………………………….  †`qv nqwb Not given…………………………………………………..  Rvbv †bB Don’t know………………………………………………. | 1  2  3  9 |  |
| 25. | wPwKrmvi djvdj wK wQj?  What was the outcome of the treatment? | m¤ú~Y© my¯’ n‡q wM‡qwQj Recovered…………………...............................  wKQzUv DbœwZ n‡qwQj Improved……………………………………….…  Ab¨ nvmcvZv‡j ¯’vbvšÍi Kiv n‡qwQj Referred to other hospital……….…  †Kvb DbœwZ nqwb No improvement……………….................................  G‡Lv‡bv wPwKrmvaxb Still under treatment…………….………………… | 1  2  3  4  5 |  |
| 26. | wPwKrmvi Rb¨ me©‡gvU KZ UvKv LiP n‡qwQj ?  How much did it cost for the treatment (Taka)? | \| Wv³vi wd Consultation fee……. \|  \| \| --- \| --- \| \| j¨v‡eiUwi cixÿv LiP…………..… Laboratory investigation cost  †eW wd Bed fee………………..... \|  \| \| Acv‡ikb LiP……………............  Operation cost \|  \| \| JlacÎ Medicine cost………...... \|  \| \| †ivMxi mvnvh¨Kvixi _vKvi LiP…….  Attendant’s accommodation cost \|  \| \| hvZvqvZ Transport cost……………. \|  \| \| Ab¨vb¨ Others…………………....... \|  \| \| **me©‡gvU Total**……………………… \|  \| | |  |
| 27. | Bb&&Rywii d‡j †Kvb c½yZ¡ n‡q‡Q wK?  Has the person been injured person become disabled? | nu¨v Yes……………………………………………..............................  bv No…………………………………………………………………..  Rvbv †bB Don’t know……………..................................................... | 1  2  9 | Q30  Q30 |
| 28. | DËi nu¨v n‡j, Zv wK ai‡bi c½yZ¡ ?  If yes, type of disability? | `„wó kw³ bó n‡q †M‡Q Loss of vision…………………………………….  kªeY kw³ bó n‡q †M‡Q Loss of hearing…………….............................  nuvUvi kw³ bó n‡q †M‡Q Loss of mobility……………………………....  nvZ w`‡q KvR Kivi kw³ bó n‡q †M‡Q Loss of activity with hand………..  evK kw³ bó n‡q †M‡Q Loss of speech…………………………………..  eyw× kw³ bó n‡q †M‡Q Loss of intellect…………………………………  Ab¨vb¨ (D‡jøL Kiæb) Others (Specify) _____________­­­­­­­­­­­­­­­­_____________ | A  BC  DEF  X |  |
| 29. | hw` 28 bs cª‡kœi DËi A-D nq, Zvn‡j cv‡ki †UwejwU c~iY Kiyb Does this disability affect one or both sides? | `„wó kw³ bó n‡q †M‡Q Loss of vision  GK w`‡K Unilateral……………..………….......................................  `yB w`‡K Bilateral…………………………........................................  kªeY kw³ bó n‡q †M‡Q Loss of hearing  GK w`‡K Unilateral…………….…………………………..………….  `yB w`‡K Bilateral…………………………….....................................  nuvUvi kw³ bó n‡q †M‡Q Loss of mobility:  GK w`‡K Unilateral……………………………………….…………...  `yB w`‡K Bilateral………………………………………………..……  nvZ w`‡q KvR Kivi kw³ bó n‡q  Loss of activity with hand  GK w`‡K Unilateral……………………….……………………………  `yB w`‡K Bilateral……………………………..................................... | 1  2  1  2  1  2  1  2 |  |
| 30. | Amy¯’Zvi Kvi‡b KZw`b ¯^vfvweK KvRK‡g© A‡b¨i mvnvh¨ MÖnb Ki‡Z n‡qwQj?  (gvm I w`‡b wjLyb)  For how long did s/he require assistance for activities of daily life after the injury? (Month & Days)  Rural context | nuvUv ………………………………… M M D D Walking  emv Ges weQvbv †_‡K DVv……………….. M M D D  Sitting down and getting out of bed  wmuwo †e‡q DVv………………………… M M D D  Climbing stairs (1/2 steps)  †Mvmj Kiv………………………….… . M M D D  Bathing  Uq‡jU e¨envi Kiv……………………… M M D D  Using toilet |  |  |
| 31. | Amy¯’Zvi Kvi‡b KZw`b ¯‹z‡j ev AuvP‡j ev Kg©¯’‡j Abycw¯’ZwQj ? Number of days of school/anchal/work loss? | Y Y M M D D |  |  |
| 32. | e¨w³wU wK Avcbv‡`i cwiev‡ii GKRb D‡jøL‡hvM¨ Avq DcvR©bKvix ?  Was the person a significant source of income for the family? | cÖavb DcvR©bKvix Main income earner…………………………………..…  Ab¨Zg DcvR©bKvix Z‡e cÖavb bq Major but not main................................  ‡MŠYDcvR©bKvix Minor…………………………............................................  DcvR©bKvix bb None…………………………...…………………………….  DËi w`‡Z mg_© bb Unable to answer…………………................................ | 1  2  3  4  5 |  |
| 33. | e¨w³wUi BbRywiRwbZ Amy¯’Zvi d‡j Avcbv‡`i cwiev‡i DcvR©b K‡g wM‡q‡Q wK ?  How has the family being coping with the loss in income? | nu¨v Borrow from friends…………………………………………………………..........  bvSell assets and household possession…………………………………………………………………  Rvbv †bB Beg…………………………………………………  Reduce family consumption of food  Go without other basic needs, e.g. clothing and shelter  Others | 1  2  9 |  |
|  | e¨w³wUi BbRywiRwbZ Amy¯’Zvi d‡j Avcbv‡`i cwiev‡I (Lvbvq) †Kvb Lv‡`¨i Afve n‡”Q wK ? | nu¨v…………………………………………………………….......  bv ………………………………………………………………  Rvbv †bB………………………………………………… | 1  2  9 | END |
