# Supplementary material for: Burden of Lesser-Known Unintentional Non-Fatal Injuries in Rural Bangladesh: Findings from a Large-Scale Population-Based Study
Source: Int J Environ Res Public Health. 2019 Sep 12;16(18):3366. doi: 10.3390/ijerph16183366 (PMC6766074; doi:10.3390/ijerph16183366)
Supplement: Supplementary file 1 [file ijerph-16-03366-s001.zip › injury modules/Mortality-revised_7th April.docx]

| **Saving of Lives from Drowning (SoLiD)**  **ICDDR,B & CIPRB**  **Baseline Survey/Injury Surveillance** | | | | | |
| --- | --- | --- | --- | --- | --- |
| BbRywiRwbZ g„Zz¨ dg©  **Injury Mortality Form** | | | | | |
|  | | | | | |
|  | | bvg Name | †KvW Code |  | |
| Dc‡Rjv Upazila | |  |  |  | |
| BDwbqb Union | |  |  |  | |
| eøK Block | |  |  |  | |
| MÖv‡gi bvg Name of the village | |  |  |  | |
| Lvbvi b¤^i Household Number | |  |  | / | |
| Lvbv cÖav‡bi bvg Name of Household Head | |  |  |  | |
| ZvwiL Date | |  | **Y**  **M**  **M**  **Y**  **D**  **D**DDD |  | |
| DËi`vZvi bvg I Lvbv m`m¨ b¤^i Name & household number of the respondent | |  |  |  | |
| No. | Questions | Coding Categories | |  | Skip |
| 1. | g„Z e¨w³i bvg  Name of deceased person | ___________________________________ | |  |  |
| 2. | g„Z e¨w³i Lvbv m`m¨ b¤^i  Deceased Person Number |  | |  |  |
| 3. | g„Z e¨w³i wj½  Sex of deceased person | cyiæl Male……………………………………………………………….  gwnjv Female………………………………………………………........ | | 1  2 |  |
| 4. | AwfcÖvq Intent  Bb&&Rywii AwfcÖvq wK wQj?  What was the intent of the injury? | Awb”QvK…Z Unintentional………………………………………………….  B”QvK…Z wb‡Ri ¶wZ Intentional/Self harm………………………………...  mwnsmZv Assault/Violence…………………………………………….....  wbY©q Kiv hvqwb Undetermined………………………………………....... | | 1  2  3  4 |  |
| 5. | ¯’vb Place  BbRywi NUvi mgq e¨w³wU †Kv_vq wQj?  Where was the person when s/he was injured? | †kvqvi Ni Bedroom……………………………………………………….  emvi Ni Living room……………………………………………………..  ivbœv Ni Kitchen…………………………………………………………..  †Mvmj Lvbv/cvqLvbv Bathroom…………………………………………….  evoxi DVvb Yard……………………………………………………..........  eviv›`v Veranda.........................................................................................  GK K¶ wewkó Ni One room dwelling …………………………………..  †kªbxK¶ Classroom.....................................................................................  ¯‹z‡ji †Ljvi gvV School playground………………………………............  wk¶v cÖwZôv‡bi †nv‡÷j Hostel of educational institute……………………  Ab¨ †Ljvayjvi ¯’vb/ µxov½b Other playground…………………………...  iv¯Zvq /gnv-mo‡K Roads/highway……………………………………….  †ij †ókb/†dix/jÂ NvU/evm÷¨vÛ/Ab¨ cwienbGjvKv…………………….....  Railway station/Ferry/ Launch station/Bus stand/ Other vehicles area  K…wl‡¶Î/Lvgvi evox/PvZvj Agricultural field ………………………………  wkí/KjKviLvbv/IqvK©kc Industry/factory/workshop……………………..  Rjvkq Water reservoir……………………………………………………  nvU/ evRvi Market/haat/bazaar…………………………………………..  Awdm Office………………………………………………………………  wbg©vY GjvKv Construction area…………………………………………….  Ab¨vb¨ (D‡jøL Kiæb) Others (Specify)____________________________  Rvbv †bB Don’t know………………………………………………….…. | | 1  2  3  4 5  6  7  8  9  10  11  12  13  14  15  16  17  18  19  20  99 |  |
| 6. | KZ Zvwi‡L BbRywi N‡UwQj ?  What was the date of injury ? | D D M M Y Y | |  |  |
| 7. | BbRwyi KLb N‡UwQj?(24N›Uvq wjLyb)  What was the time of injury?  (in 24 hours) | H H M M | |  |  |
| 8. | e¨w³wUi (bvg D‡jøL Kiyb) K‡e g„Zy¨ n‡qwQj? When (date) did the person die? | D D M M Y Y | |  |  |
| 9. | e¨w³wUi (bvg D‡jøL Kiyb) KLb g„Zy¨ n‡qwQj? (24 N›Uvq wjLyb)  When (in 24 hours) did the person die? | H H M M | |  |  |
| 10. | e¨w³wUi (bvg D‡jøL Kiyb) wK NUbv¯’‡jB g„Zz¨ n‡qwQj?  Did the person die on the spot? | n¨vu Yes………………………………………………………………...…  bv No…………………………………………………………………..…..  Rvbv †bB Don’t know………………………………………………….….. | | 1  2  9 |  |
| 11. | g„Zz¨i ¯’vb:  Place of death  †Kvb RvqMvq gviv wM‡qwQj?  Where did the person die? | nvmcvZv‡j Hospital………………………………………………….…..  wbR evox‡Z Home…………………………………………………….…..  iv¯Ívq On the road……………………………………………….………  evox‡Z †bIqvi c‡_ Way to home……………………………….……….  nvmcvZv‡j †bIqvi c‡_ Way to hospital………………………….………  Ab¨vb¨ (D‡jøL Kiæb) Others (Specify)…………….………………………. | | 1  2  3  4  5  6 |  |
| 12. | c×wZ Injury Mechanism  BbRywi c×wZ wK wQj? What was the external cause of injury?  wbw`©ó BbRywi c×wZ dg©c~iY Kiæb  Please fill in the specific injury mechanism form | AvZ¥nZ¨v-Gg 1 Attempt to suicide/suicide-M1……………………………  moK `yN©Ubv-Gg 2 Transport injury- M 2…………………………………  mwnsmZv -Gg 3 Violence-M 3…………………………………………….  c‡o hvIqv -Gg 4 Fall-M 4……………………………………………….  aviv‡jv e¯‘ Øviv †K‡U hvIqv-Gg 5 Cut injury-M 5………………………...  cy‡o hvIqv-Gg 6 Burn-M 6……………………………………………...  Wy‡e hvIqv-Gg 7 Drowning-M 7…………………………………………..  `yN©UbvRwbZ welcvb-Gg 8 Accidental poisoning -M 8……………………..  †gwkb/hš¿cvwZi AvNvZ -Gg 9 Machine injury-M 9………………………..  we`~¨r¯ú„„ó -Gg 10 Electrocution-M 10…………………………………...  cÖvbx I KxU cZ‡½i Kvgo / AvNvZ-Gg 11 Animal injury-M 11…………….  †fuvZv e¯‘i AvNvZ -Gg 13 Blunt object-M 13……………………………...  `yN©Ubv RwbZ k¦vm‡iva-Gg 14 Suffocation-M 14…………………………….  cošÍ e¯‘i AvNvZ Falling object…………………………………………….  Ab¨vb¨ (D‡jøL Kiæb) Others (Specify) ____________________ ______  Rvbv †bB Don’t know………………………………………………… | | 1  2  3  4  5  6  7  8  9  10  11  13  14  15  16  99 |  |
| 13. | ¶Z A½ I BbRywii aiY:  (Injured parts and types of injury)  e¨w³wUi kix‡ii †Kvb& †Kvb& A‡½ Ges  wK ai‡bi BbRywi n‡qwQj?  What were the injured parts and the type of injury? |  | |  |  |

|  | ¶Z A½ Injured parts BbRywii aiY Injury types (Main three) | | | | | | | | | |
| --- | --- | --- | --- | --- | --- | --- | --- | --- | --- | --- |
|  | 1. gv_v Head |  |  |  |  |  |  |  |  |  |
|  | 2. gyLgÛj Face |  |  |  |  |  |  |  |  |  |
|  | 3. Nvo Neck |  |  |  |  |  |  |  |  |  |
|  | 4. eyK/wcV Chest |  |  |  |  |  |  |  |  |  |
|  | 5. †cU Abdomen |  |  |  |  |  |  |  |  |  |
|  | 6. evû nvZ e¨ZxZ Upper extremity (except hand) |  |  |  |  |  |  |  |  |  |
|  | 7. nvZ Hand |  |  |  |  |  |  |  |  |  |
|  | 8. wbgœv½ (cv‡qi cvZv e¨ZxZ) Lower extremity (except foot) |  |  |  |  |  |  |  |  |  |
|  | 9.cv‡qi cvZv Foot |  |  |  |  |  |  |  |  |  |
|  | 10.†Kvgi Waist |  |  |  |  |  |  |  |  |  |

**BbRywi ai‡bi †KvW (Injury types):** 01. nvo fv½v (Fracture) 02.gP‡K hvIqv (Sprain) 03. nvo m‡i hvIqv (Dislocation) 04. †K‡U hvIqv / Db¥y³ ¶Z(Cut/open wound) 05. Kvgo (Bite) 06.wQ‡j/ †_uZ‡j hvIqv (Abrasion/laceration) 07.cy‡o hvIqv (Burn.) 08.gv_vq AvNvZ (Head injury) 09. kix‡ii wfZ‡ii A‡½i ¶Z (Internal injury/internal organ injury) 10. Ab¨vb¨ (D‡jøL Kiæb) (Others specify)………………………

| 14. | hLb BbRywi N‡UwQj ZLb e¨w³wUi Ae¯’v †Kgb wQj? What was the condition of the victim just after injury? | mÁvb Conscious……………………………………………..........  msÁvnxb Unconscious………………………………………………  Rvbv †bB Unknown………………………….................................. | 1  2  9 | Q16  Q16 |
| --- | --- | --- | --- | --- |
| 15. | hLb BbRywi N‡UwQj, ZLb e¨w³wUi Pjv‡div Kivi ¶gZv †Kgb wQj? (hw` Ávb _v‡K)  What was the mobility condition of the person just after injury (if answer is conscious) | GKv GKv nuvU‡Z †c‡iwQj Mobile alone…………………………………..  A‡b¨i mn‡hvwMZvq nuvU‡Z †c‡iwQj Mobile with assistance……………….  nvuU‡Z cviwQj bv Immobile……………………………………………….  Rvbv †bB Don’t know………………………………………………….. | 1  2  3  9 |  |
| 16. | e¨w³wU‡K †KD wK cÖv_wgK wPwKrmv w`‡qwQj?  Did the person receive first aid? | nu¨v Yes…………………………………………………………………..  bv No……………………………………………………………………  Rvbv †bB Don’t know…………………………………………………… | 1  2  9 | Q19  Q19 |
| 17. | DËi nu¨v n‡j, †K cÖv_wgK wPwKrmv w`‡qwQj? (GKvwaK DËi)  If yes, who gave the person first aid? (Multiple response) | gv Mother…………………………………………………….................  evev Father ………………………………………………………………  ¯^vgx-¯¿x Husband/wife…………………………………………………..  dv‡g©mx/cjøx wPwKrmK Medicine shopkeeper/village doctor………………  Ab¨ cÖvß eq¯‹ †mev`vbKvix Other adult caregiver…………………………  fvB/†evb Brother/sister……………………………………………..........  eÜz / mgeqmx wkï Friend/peer……………………………………………  cÖwZ‡ekx Neighbor………………………………………………………  gvV ch©v‡qi ¯^v¯’¨Kg©x Community health worker………………………….  KwgDwbwUi †¯^”Qv‡mex Community volunteer………………………………  Wv³vi/nvmcvZvj/wK¬wbK Doctor/hospital/clinic……………………………  wb‡RB Himself/herselfOwn……………………………………………  Ab¨vb¨ (D‡jøL Kiæb) Others (specify) ____________________________ | A  B  C  D  E  F  G  H  I  J  K  L  X |  |
| 18. | †m wK cÖv_wgK wPwKrmvq cÖwk¶Y cÖvß ?  Was s/he trained in first aid? | nu¨v Yes…………………………………………………………….........  bv No……………………………………………………………………  Rvbv †bB Don’t known………………………………………………… | 1  2  9 |  |
| 19. | BbRywii Rb¨ †Kvb wPwKrmv MÖnb Kiv n‡qwQj wK ? Did the person receive treatment for injury? | nu¨v Yes……………………………………………………………..........  bv No……………………………………………………………………  Rvbv †bB Don’t know…………………………………………………… | 1  2  9 | Q30  Q30 |
| 20. | wPwKrmv cÖ`vbKvix  Treatment provider  AvnZ e¨w³‡K †K wPwKrmv w`‡qwQj?  (GKvwaK DËi)  Who provided the treatment?  (Multiple response) | †iwRóvW© Wv³vi Registered doctor…………………………………………….…...  †gwW‡Kj Gwm÷¨v›U Medical Assistant/SACMO ………………………………..  ¯^v¯’¨ mnKvix / cwievi Kj¨vY cwi`wk©Kv / cwievi Kj¨vY mnKvix…………………  Health Assistant/Family Welfare Visitor/ Family Welfare Assistant  Gb. wR. I ¯^v¯’¨ Kg©x NGO Service Provider……………………..……………..  Jl‡ai †`vKvb`vi / cjøx wPwKrmK…………………………………………………….  Medicine shopkeeper/Village doctors  †nvwgIc¨vw_K wPwKrmK Homeopathic Practitioner………………………..…….  KweivR Herbal Medicine Practitioner……………………………………...........  cÖwk¶Y cÖvß `vB Trained TBA……………………………………….……………...  Svo duyKKvix / Bgvg/cy‡ivwnZ Traditional healer/Religious leader…..………  AvZ¥xq-¯^Rb / eÜz-evÜe Relative/Friends…………………………………………  Ab¨vb¨ (D‡jøL Kiæb) Others (Specify)____________________________ | A  BC  D  E  F  G  H  I  J  X |  |
| 21. | †mev`vbKvix cÖwZôvb  Service provider  e¨w³wU †Kv_vq wPwKrmv wb‡qwQj?  (GKvwaK DËi)  Where did the person receive treatment?  (Multiple answer) | we‡klvwqZ nvmcvZvj (†gwW‡Kj K‡jR nvmcvZvj, c½y nvmcvZvj, wc. wR. …… nvmcvZvj BZ¨vw`) Specialized hospital (Medical College Hospital, Orthopaedic Hospital, Post graduate Hospital etc  †Rjv nvmcvZvj District Hospital…………………………………………  Dc‡Rjv ¯^v¯’¨ Kg‡cø· Upazila Health Complex………………………….  BDwbqb ¯^v¯’¨ I cwievi Kj¨vY †K›`ª ……………………………………..  Union Health and Family Welfare Centre  cÖvB‡fU wK¬wbK Private Clinic……………………………………….........  Gb.wR.I wK¬wbK NGO Clinic……………………………………….........  †mev`vbKvixi cÖvB‡fU †P¤^vi Private practitioners’ chambers………...........  dv‡g©mx/Jl‡ai †`vKvb`vi Pharmacy/medicine shop………………………  wbRevox Own home……………………………………………………...  Ab¨vb¨ (D‡jøL Kiæb) Others (Specify)_____________­­­­­­­­­­­______________ | A  B  C  D  E  F  G  H  I  X |  |
| 22. | e¨w³wU nvmcvZv‡j/¯^v¯’¨†K‡›`ª fwZ© n‡qwQj wK bv?  Was the injured person admitted to a health facility? | nu¨v Yes…………………………………………………………….........  bv No……………………………………………………………………  Rvbv †bB Don’t know…………………………………………………… | 1  2  9 | Q25  Q25 |
| 23. | (DËi nu¨v n‡j) e¨w³wU hw` nvmcvZvj / ¯^v¯’¨‡K›`ª /wK¬wb‡K fwZ© n‡q _v‡K Zvn‡j †Kvb ai‡bi nvmcvZv‡j ?  What type of health facility was s/he admitted to? | we‡klvwqZ nvmcvZvj (†gwW‡Kj K‡jR nvmcvZvj, c½y nvmcvZvj, wc. wR. nvmcvZvj BZ¨vw`) Specialized hospital (Medical College Hospital, Orthopaedic Hospital, Post graduate Hospital etc…………………......  †Rjv nvmcvZvj District Hospital………………………………………..  Dc‡Rjv ¯^v¯’¨ Kg‡cø· Upazila Health Complex…..……………………..  cÖvB‡fU wK¬wbK Private Clinic………………………………………........  Gb.wR.I wK¬wbK NGO Clinic…………………………………………….  Ab¨vb¨ (D‡jøL Kiæb) Others (Specify)__________________________ | 1  2  3  4  5  9 |  |
| 24. | e¨w³wU‡K nvmcvZv‡j/†mev`vbKvixi wbKU wKfv‡e wb‡q hvIqv n‡qwQj ?  (GKvwaK DËi)  Transportation to hospital/other treatment provider:  How was the patient transported to the health facility/or healthcare provider?  (Multiple responses) | G¤^y‡jÝ Ambulance………………………………………………………  Ab¨ †Kvb †gvUi PvwjZ Mvwo‡Z (evm, Rxc, Kvi Ges wZb PvKvwewkó Mvwo) Other motorized vehicle (bus, jeep car and three wheeler…………..…  †gvUinxb hvbevnb (wiKkv, wiKkv f¨vb, †VjvMvwo) Non-motorized vehicle (rickshaw, rickshaw van, cart etc…)……………………………….…..  †bŠKv Boat………………………………………………………..…….  †dix /jÂ /÷xgvi Ferry/launch/steamer…………………………...….…  †mev`vbKvix AvNvZcÖvß e¨w³‡K evwo‡Z †`L‡Z G‡mwQ‡jb…………..…….…  Treatment provider visited the injured person at home  Ab¨vb¨ (D‡jøL Kiæb) Others (Specify) __________________________  Rvbv †bB Don’t know……………………………………………...…... | A  B  C  D  E  F  G  X |  |
| 25. | AvNvZ cvIqvi ci nvmcvZv‡j/†mev`vbKvixi wbKU †cuŠQv‡Z KZ mgq †j‡MwQj ? (N›Uvq wjLyb) [hw` GK N›Uvi Kg nq Z‡e 000 wjLyb]  How much time did it take to reach the provider? (in hours) [if less than one hour write 000] |  |  |  |
| 26. | e¨w³ nvmcvZv‡j KZw`b fwZ© wQj ?  How many days was the person admitted in the hospital? |  |  |  |
| 27. | mvR©vix ev Acv‡ikb †j‡MwQj wK ?  Was surgery/operation done? | nu¨v Yes……………………………………………………………..........  bv No…………………………………………………………………….  Rvbv †bB Don’t know…………………………………………………… | 1  2  9 | Q29  Q29 |
| 28. | hw` n¨vu nq, Zvn‡j wK ai‡bi A¨v‡bm&‡_wmqv †`qv n‡qwQj?  If yes, what type of anesthesia was given? | †jvKvj Local…………………………………………………………….  mvaviY General……………………………………………………..........  †`qv nq wb Not given…………………………………………………….  Rvbv †bB Don’t know………………………………………………….. | 1  2  3  9 |  |
| 29. | wPwKrmvi Rb¨ me©‡gvU KZ UvKv LiP n‡qwQj ?    How much did it cost for the treatment (Taka)? | \| Wv³vi wd Consultation fee……. \|  \| \| --- \| --- \| \| j¨v‡eiUwi cixÿv LiP…………..… Laboratory investigation cost  †eW wd Bed fee………………..... \|  \| \| Acv‡ikb LiP……………............  Operation cost \|  \| \| JlacÎ Medicine cost………...... \|  \| \| †ivMxi mvnvh¨Kvixi _vKvi LiP…….  Attendant’s accommodation cost \|  \| \| hvZvqvZ Transport cost……………. \|  \| \| Ab¨vb¨ Others…………………....... \|  \| \| **me©‡gvU Total**……………………… \|  \| |  |  |
| 30. | `yN©UbvwU wK _vbvq Rvbv‡bv n‡qwQj?  Was the event reported to the police? | nu¨v Yes……………………………………………………………..........  bv No…………………………………………………………………….  Rvbv †bB Don’t know…………………………………………………… | 1  2  9 | Q32  Q32 |
| 31. | g„Z e¨w³i gqbv Z`¤Í Kiv n‡qwQj wK?  Was a post-mortem done? | nu¨v Yes…………………………………………………………………..  bv No…………………………………………………………………....  Rvbv †bB Don’t know………………………………………………..…. | 1  2  9 |  |
| 32. | e¨w³wU wK Avcbv‡`i cwiev‡ii GKRb D‡jøL‡hvM¨ Avq DcvR©bKvix ?  Was the person a significant source of income for the family? | cÖavb DcvR©bKvix Main income earner………………………………..….  Ab¨Zg wKš‘ cÖavb bq Major but not main……………………………..…  †MŠY Minor……………………………………………………………....  DcvR©bKvix bb None………………………………………………….…  Rvbv †bB Don’t know………………………………………………..… | 1  2  3  4  9 |  |
| 33. | e¨w³wUi g„Z¨yi d‡j Avcbv‡`i cwiev‡ii DcvR©b K‡g wM‡qwQj wK ?  Due to the death of the person was there any decline in outsource income in your family? | nu¨v Yes……………………………………………………………..........  bv No…………………………………………………………………….  Rvbv †bB Don’t know…………………………………………………… | 1  2  9 |  |
| 34. | e¨w³wUi g„Z¨yi d‡j Avcbv‡`i cwiev‡ii Lv‡`¨i Afve n‡q‡Q wK ?  Due to the death of the person was there any decline in food consumption in your family? | nu¨v Yes……………………………………………………………..........  bv No…………………………………………………………………….  Rvbv †bB Don’t know………………………………………………..… | 1  2  9 |  |
|  | g„Z¨yi †ÿ‡Î NUbvi mswÿß weeiY  If death describe what happened | ..................................................................................................................  ……………………………………………………………….……………………………………………………….………….………………..  ………………………………………………….….…………………..  …………………………………………………………….……………. |  | END |
